# On fault-tolerant Boolean functions in proteinoids–ZnO colloids

**DOI:** 10.1007/s10854-025-14302-3

**Published:** 2025-02-02

**Authors:** Noushin Raeisi Kheirabadi, Panagiotis Mougkogiannis, Raphael Fortulan, Nic Roberts, Alessandro Chiolerio, Andrew Adamatzky

**Affiliations:** 1 Unconventional Computing Laboratory, UWE, Bristol, UK; 2 Department of Engineering and Technology, University of Huddersfield, Huddersfield, UK; 3 Center for Bioinspired Soft Robotics, Istituto Italiano di Tecnologia, Genoa, Italy

## Abstract

This study investigates the computational properties of ZnO colloids in combination with proteinoid microspheres within an unconventional computing framework. We propose a method for creating flexible and fault-tolerant logic gates utilising this colloidal system. The colloidal matrix receives binary strings with an electrical impulse representing a logical “True” and its absence representing a “False”. Electrical responses are recorded, and Boolean functions are extracted. This nano-bio hybrid of ZnO colloids and proteinoids has the potential to power next-generation unconventional computing systems that can adapt to changing environments, paving the way for novel nano-bio hybrid computing architectures.

## Introduction

There has been a notable evolution in the field of unconventional computing [1–3]. A historical example would be the hydraulic algebraic machines [4–6], liquid computing [7], which includes the most recent analysis of neuromorphic architectures implementable in colloids [8–10], and hydraulic mathematical machine integrators, fluid mappers, fluidic logic devices, liquid marble computers, and reaction-diffusion systems [11–13]. Expanding on these bases, our study investigates the computational limits of colloidal mixtures, in particular the combination of proteinoids and ZnO nanoparticles.

Liquid cybernetic systems, also known as colloidal autonomous soft holonomic processors, have shown promising autolographic capabilities [14, 15]. Our earlier controlled lab work with ZnO colloids demonstrated their potential as electrical-analogue neurones, effectively executing synaptic-like learning and Pavlovian reflexes [16, 17]. Furthermore, $\text{Fe}_{3}\text {O}_{4}$ Fe_3_O_4_ ferrofluid’s computational capabilities for digit recognition provided additional evidence of the adaptability of liquid-based systems [18].

We study the unique combination of ZnO colloids and proteinoids in colloidal mixtures. ZnO, recognised for its non-toxic biocompatibility and varied semiconductor applications [19–26], could be a suitable choice for unconventional computing. Controlled tests reveal that ZnO colloids are not only models of electrical-analogue neurones but also substrates for Boolean logic implementation, as demonstrated by using binary strings and a systematic classification of electrical output responses [27, 28].

Proteinoids, which resemble proteins and emerge under simulated prebiotic settings, have sparked interest in origin-of-life studies [29]. Recent research demonstrates their computational abilities in liquid solutions [30]. These results provide important insight into prebiotic chemistry and possible applications in biomolecular computing by indicating that proteinoids may have been involved in basic information processing prior to the origin of living beings [31–34].

To achieve sufficient recognition accuracy in neuromorphic computing applications, error detection and tolerance methods must be implemented. As a result, substantial research on fault detection and tolerance has been done in recent years, with a focus on RRAM-based computing systems (RCS) [35–37].

Recent advances in neuromorphic architectures, fluidic logic devices, and liquid computing have expanded the possibilities of computation beyond traditional digital models. This study contributes to the field of colloidal mixtures by studying the computational effects of colloidal mixtures that combine ZnO nanoparticles and proteinoid microspheres. While proteinoids, which are synthetic molecules that resemble proteins, demonstrate computational skills that have implications in both prebiotic chemistry and biomolecular computing, zinc oxide (ZnO) is suited for unconventional computing due to its biocompatibility and semiconductor qualities. Here, by utilising these properties to create fault-tolerant logic gates in a hybrid system, we present a framework for dynamic, reconfigurable computing in nano-bio hybrid systems.

## Experimental details

### Synthesis

ZnO nanoparticles were prepared by Plasmachem company. Dimethyl sulfoxide (DMSO), Dr. Moran’s brand pharmaceutical Grade 99.9%, was purchased from Amazon. Sodium dodecyl sulphate (SDS) and sodium hydroxide (NaOH) were purchased from Merck. De-ionised water (DIW) was prepared in the lab with a Millipore de-ionised water generator device, model Essential, rated at 15 M$\Omega$ cm. A colloid suspension of ZnO nanoparticles in DMSO with a concentration of 0.3 mg/ml was prepared according to a method described elsewhere [16, 38].

**Fig. 1:**
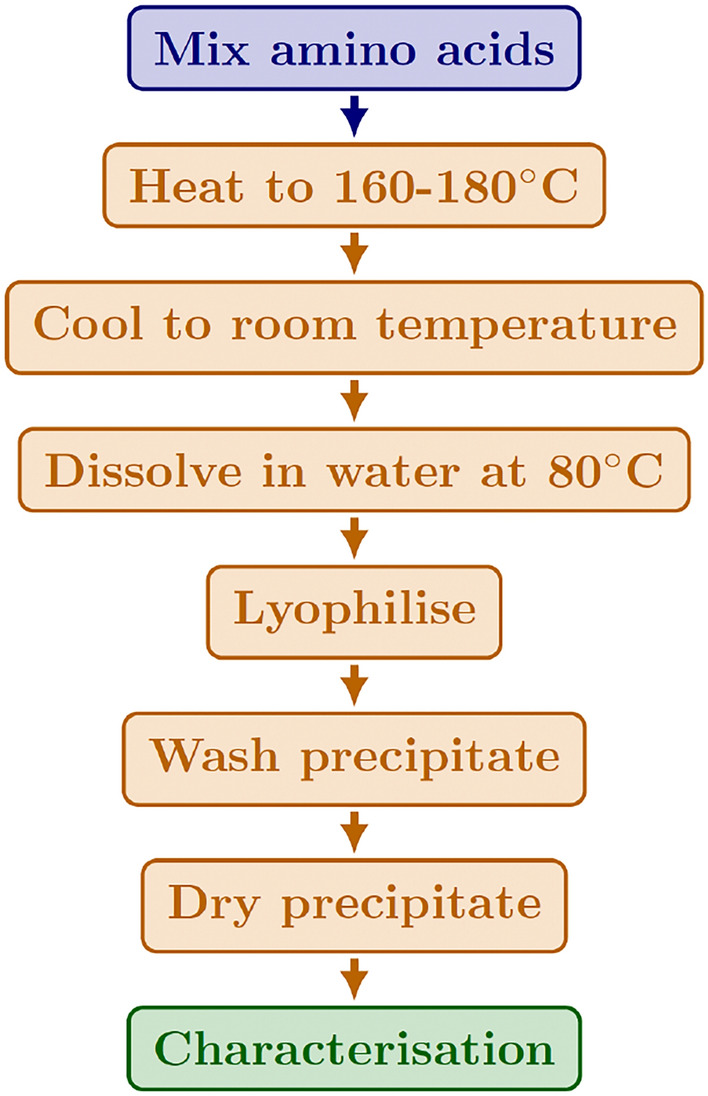
proteinoids synthesis process

l-Glutamic acid and l-aspartic acid were purchased from Sigma Aldrich at a purity of 98%. Proteinoids were synthesised using procedures outlined in the reference [39]. A reaction flask containing glycine, alanine, aspartate, and glutamate is well mixed with amino acids to start the production of proteinoids. Thermal polymerisation is then applied to the amino acid combination, with the flask being heated to temperatures that usually fall between 160 and 180 °C. The higher temperatures make it easier for the amine and carboxyl groups in the amino acids to initiate the condensation processes. Peptide bonds so develop to join amino acids to form polypeptide chains of varying lengths. The polymerisation reaction is stopped by cooling the flask after the heating process. The mixture is then allowed to cool before being dissolved in 80 °C hot water. The produced proteinoids precipitate out of the aqueous solution. The precipitate is freeze-dried during the lyophilization process, which is the next step in separating the proteinoids from the water. The ultimate product, proteinoids, is obtained by thoroughly drying the solid precipitate after it has undergone washing operations to eliminate impurities. To summarise, this procedure makes it possible to create proteinoids from basic amino acid building blocks [39]. As illustrated in Fig. 1, the proteinoid production methodology entails several processing steps, including heating, dissolving, and lyophilization.

The ZnO colloids and proteinoids mixture (P-ZnO) was produced by combining equal amounts of each ingredient.

### Materials characterisation

Field emission scanning electron microscopy (FEI Quanta 650 FESEM) was employed for the characterisation of nanoparticle suspension, proteinoids, and also a mixture of proteinoids+ZnO colloids. Adjustment of the images’ contrast and brightness was performed to optimise visibility, ensuring clear differentiation between particles and the background.

The absorbance of the samples at room temperature was measured using an Ultraviolet–Visible (UV–Vis) spectrometer, specifically the Perkin Elmer Lambda XLS instrument. Dynamic Light Scattering (DLS) measurements were conducted to determine the z-average hydrodynamic diameter with a Zetasizer Nano ZS (1000 HS, Malvern Instrument Ltd., UK).

A Fourier-transform infrared spectrometer (FTIR, Thermo Scientific) was employed to characterize the proteinoids, capturing spectra over a range of 400 to 4000 cm^−1^ with a resolution of 4 cm^−1^. Data collection and spectrum analysis were conducted using the Bicolet Omnic software (OMNIC Series Software, Thermo Scientific) following FTIR spectroscopy.

### Extracting Boolean gates

The extraction of the logical gates was performed using an in-house built device. The hardware was based around an Arduino Mega 2560 (Arduino, China) and a series of programmable signal generators, AD9833 (Analog, USA). The strings were encoded following the bipolar return-to-zero logic, with the logical 0 being encoded as − 5 V and logical 1 being encoded as 5 V. A series of 2, 4, 8, Platinum/Iridium input electrodes of $10\,{\upmu }\text {m}$ diameter was placed in the mixture with a separation of ~ 5 mm to extract 2, 4, and 8-bit logical gates circuits, respectively. A set of two Platinum/Iridium electrodes of $10\,{\upmu }\text {m}$ with a separation of ~ 5 mm electrodes was then placed in parallel to measure the output potential. The output electrodes were connected to a 24-bit A/D converter (ADC-24, PICO Technology, UK). The 3rd channel was used to pass a pulse to the ADC on every input state change.

**Fig. 2:**
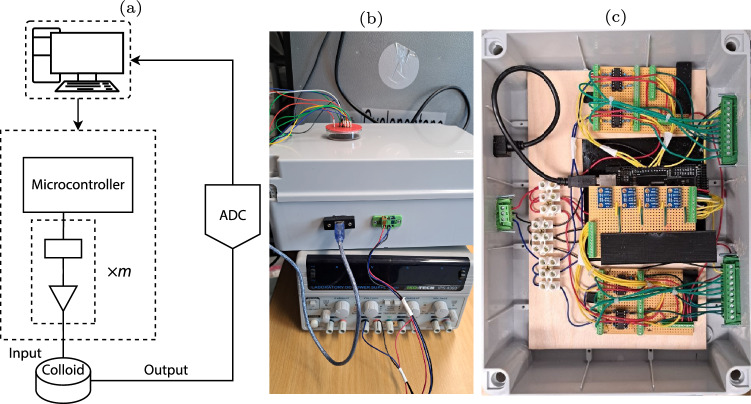
**a** The logic circuit extraction scheme involves the integration of an ADC. Adapted from [40]. **b** picture of the experimental setup. **c** Photo of the hardware inside the box

A PC is used to programme a Control Unit (CU) and receive the readings from an analogue-to-digital converter (ADC), as shown in Fig. 2a. The Arduino Mega and several AD9833 ICs are housed in the CU, which is shown in Fig.2c as the grey box linked to a typical laboratory power supply. The programmable signal generators are utilised to generate the negative voltage signals since the target output is within the range of [− 5,5] V, while the Arduino boards’ outputs vary from 0 to 5 V.

A sequence of binary strings $b = \lbrace 0,1\rbrace ^m$, *m* = 2, 4, and 8, was applied into the colloid mixture by changing every 15 s for *m* = 2, 4, and 8 and the output potential was sampled at a frequency of 1 Hz. There were a total of 67 repeats (Fig. 2). A sequence of two, four, or eight-bit strings was sent into the colloid, with a state change occurring every 15 s, counting up from binary *00* to *11*, *0000* to *1111* or *00000000* to *11111111*. In other words, every scenario conceivable for the accessible electrodes was examined. Every 15 s, the inputs’ states are successively changed for the two-bit scenario, ranging from 00 to 11. Similarly, the sample is subjected to each potential state of the four- and eight-bit strings.

## Results and discussion

### Structural characterisation

**Fig. 3:**
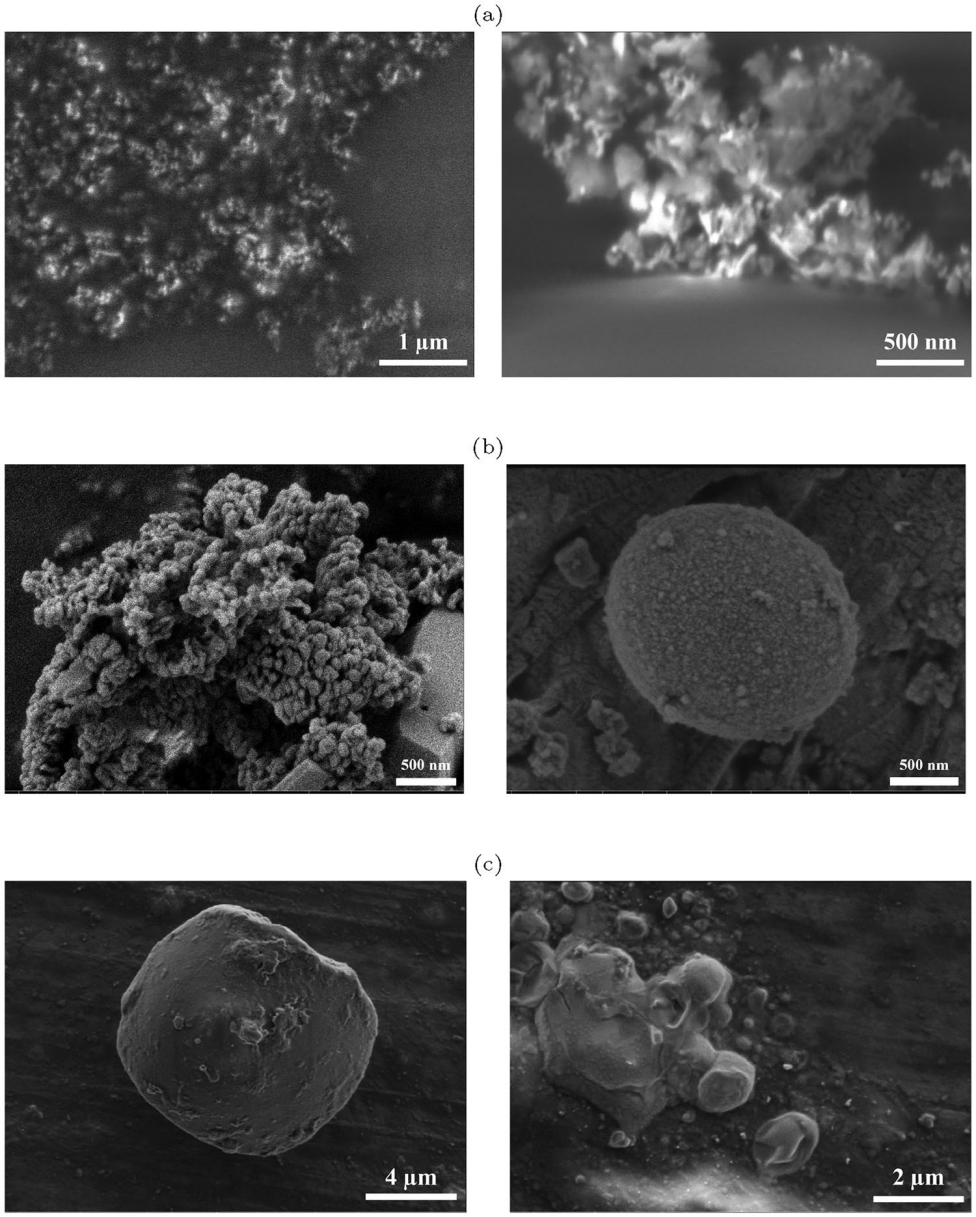
**a** FESEM images of drop cast ZnO colloids on the copper substrate. Adapted from [17]. There are observable signs of ZnO nanoparticle aggregation and clustering caused by solvent evaporation. **b** FESEM images of proteinoid microspheres developed under different ionic strength conditions. The left picture shows a prototypical proto-brain structure, with its characteristic morphology and surface topology formed in the absence of salt. Conversely, the image on the right reveals a considerably larger microsphere, the result of increasing the ionic strength during the self-assembly process via added NaCl. Adapted from [41]. **c** The scanning electron micrographs indicate the potential interaction between proteinoids and ZnO nanoparticle (NP) assemblies characterised by significant interface clustering

A thin layer of the mixture of ZnO colloids and proteinoids (P-ZnO) was prepared to analyse the particles’ morphology and size using the FESEM (Field-Emission Scanning Electron Microscopy) technique. This was done by drop-casting a drop of the mixture onto a Copper foil with a 200 μm thickness. The preparation was carried out at room temperature. The FESEM results, as shown in Fig. 3a, disclose the presence of ZnO nanoparticle agglomeration during SEM sample preparation. Due to the surface tension of the evaporating solvent, most ZnO spheres exhibit agglomeration and a multilayered structure. Notably, the pre-analysis drying process appears to induce re-aggregation of the nanocrystals. This is evidenced by the layered, network-like structures observed in the microscopy images. These structures are likely a consequence of interfacial surface tension and capillary forces, rather than a true representation of the isolated nanocrystals [42]. A cluster of proteinoids is depicted in SEM picture Fig. 3b, highlighting their morphology and texture. ZnO nanoparticles are seen to be clustered on the surfaces of proteinoid structures in the SEM images shown in Fig. 3c.

The heterostructure characteristics seen in this investigation are significantly influenced by the orienting ability of ZnO nanoparticles under an applied electric field. The alignment of ZnO particles, influenced by their intrinsic dipole moments, creates anisotropic features that can directly affect the system’s stability, functionality, and interaction dynamics. These effects are especially important in situations where orientation controls ZnO interaction with the proteinoid matrix and subsequent electrical behaviour. ZnO nanoparticles have significant dipole moments being extremely responsive to external electric fields. When an electric signal is applied, these nanoparticles align in the field direction, resulting in ordered arrangements within the colloidal suspension [16]. This alignment affects the heterostructure parameters in the following ways:

- Enhanced Particle Interaction: The orientation encourages closer packing and enhanced interparticle interactions, which can result in a reduction in free volume inside the ZnO–proteinoid mixture. This is demonstrated by the observed morphological patterns in Fig. 3 [27].

- Improved Electric Field Response: ZnO nanoparticle alignment produces anisotropy in the system’s dielectric characteristics, which improves the system’s overall sensitivity to external signals. Additionally, by stabilising dipole–dipole interactions, this alignment can lower unpredictability and increase heterostructure reproducibility [20].

In addition to influencing the colloidal suspension’s physical structure, the orienting ability also affects important characteristics such as

- *Electrical conductivity*: Better charge transport is made possible by the ZnO particles’ ordered structure because aligned particles limit charge carrier scattering paths [25].
- *Structural stability*: According to Fig. 3, orientation helps maintain the heterostructure’s integrity during electrical signal application, minimizing the effects of external disturbances [43].
- *Logic gate performance*: For fault-tolerant logic gates, by minimising random fluctuations and stabilising the dipole orientation, the orienting ability provides consistency in the system’s output [44, 45].

**Fig. 4:**
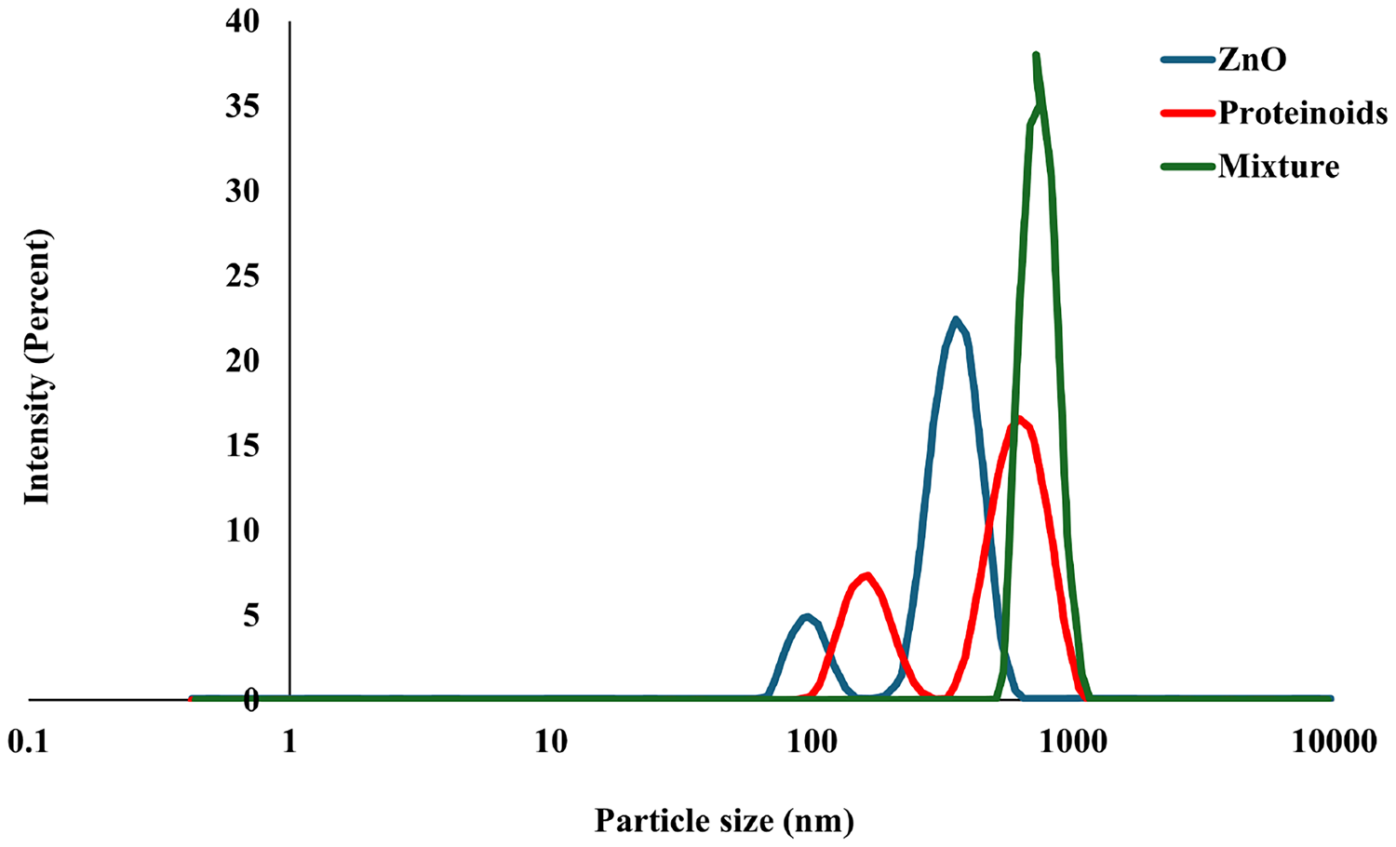
The particle size distribution and average particle size for ZnO colloidal suspension, dispersed proteinoids, and their mixture

Dynamic Light Scattering analysis was employed to determine the particle size distribution of the colloidal suspensions (Fig. 4). The average particle sizes for ZnO, proteinoids, and their mixture were found to be 361 nm, 648 nm, and 746 nm, respectively. The findings in Fig. 3c are supported by these data, which indicate that ZnO nanoparticles aggregate onto the surface of proteinoid spheres, increasing the total particle size. The interaction between particles within the colloids, driven by Brownian motion, likely facilitates electrical interactions, contributing to the observed agglomeration.

**Fig. 5:**
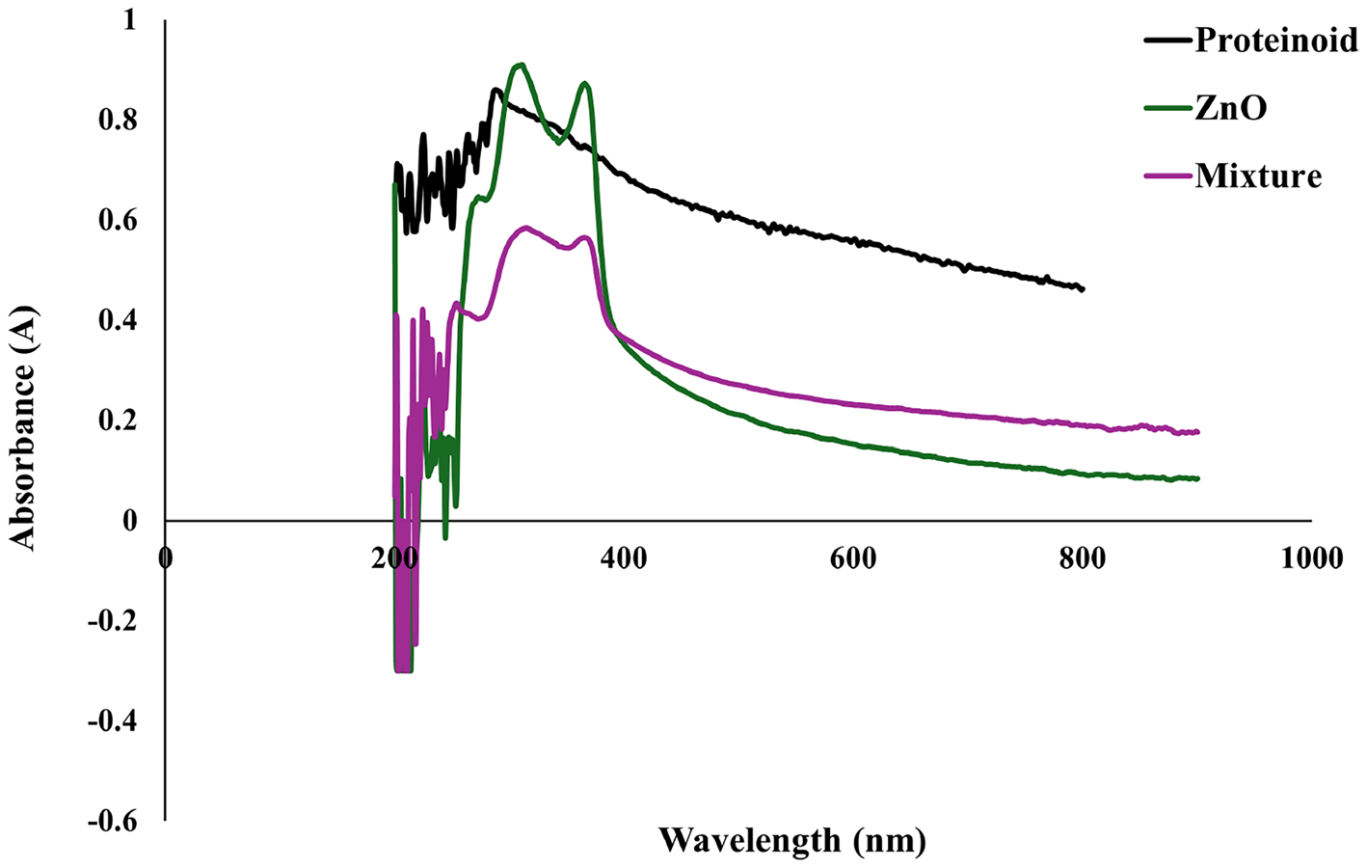
The UV–Vis absorption spectrum of the ZnO colloid, proteinoid, and their mixture

The UV–Vis absorption spectrum (Fig. 5) provides insight into the optical properties of the individual components (ZnO and proteinoid) as well as their mixture. The ZnO colloid has a signified absorption peak at 360 nm, which correlates to its characteristic UV absorption. This peak results from the electrical transition from the valence to the conduction band, which is connected with ZnO’s band gap [16]. This absorption peak is usually located between 360 and 380 nm due to ZnO’s wide band gap, which shows that the sample is well-prepared and still has the anticipated optical characteristics of ZnO nanoparticles. The absorbance steadily decreases as the wavelength approaches the visible range, indicating low absorption, which is consistent with ZnO’s transparency in visible light.

The proteinoid exhibits a distinct profile, with broad UV absorbance and a tail that extends into the visible area. The constant decrease in absorbance from UV to visible wavelengths indicates that the proteinoid contains a diversified collection of organic compounds that absorb over a wide range. This feature could be related to the existence of conjugated systems in the proteinoid structure, which interact with light throughout a wide range.

The ZnO–proteinoid mixed spectrum exhibits features found in both the ZnO and proteinoid spectra. The mixture has an absorption peak at roughly 360 nm that is similar to that of ZnO, although slightly less intense. This attenuation may imply an interaction between the ZnO nanoparticles and the proteinoid matrix, such as binding or partial aggregation. Such interactions may cause slight modifications in the optical characteristics of ZnO, as the proteinoid may influence the local electronic environment of the ZnO particles. Furthermore, the mixture exhibits a broad absorbance similar to the proteinoid spectrum, which extends into the visible range. This shows that the proteinoid matrix plays a considerable role in total absorbance in the visible range. The combined spectrum’s shape and intensity confirm that, while ZnO remains the principal absorber in the UV area, the proteinoid contributes absorbance across the entire visible spectrum, potentially improving the hybrid material’s optical functioning [27].

**Fig. 6:**
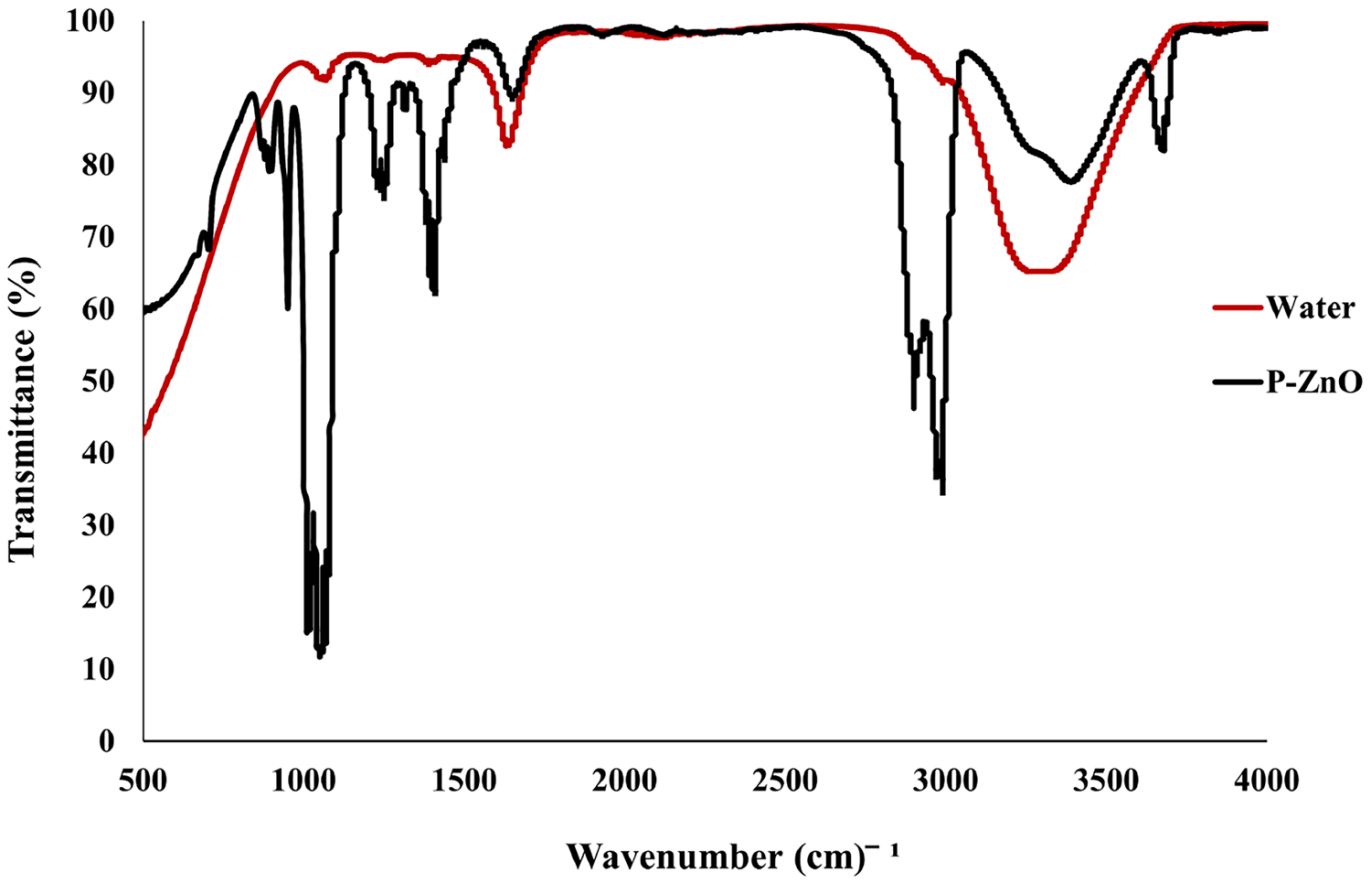
FTIR spectra of proteinoid–ZnO colloidal mixture. The spectra show Transmittance(%) as a function of Wavelength cm^−1^ for the mixture. Adapted from [43]

Figure 6 illustrates the FTIR spectra for the proteinoid–ZnO colloidal mixture, providing insights into structural characteristics across varying compositional ratios. Key spectral features include O–H and N–H stretching vibrations around ( 3300 cm^−1^), amide I and II bands within the 1500–1700 cm^−1^ range, and C–O and C–N stretching vibrations between 1000–1200 cm^−1^. Variations in these spectral features suggest notable interactions between the proteinoid and ZnO components [46].

### Logical circuits extraction

**Fig. 7:**
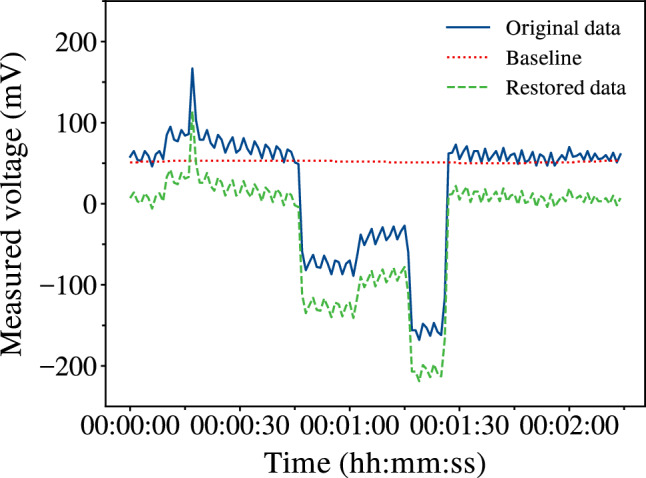
Measured output voltage of proteinoid + ZnO colloid mixture for a 2-bit binary string

The recorded potential of binary string $b = \lbrace 0,1\rbrace ^2$ is exemplified in Fig. 7. Due to the presence of external and internal factors, some of the recorded signals (as seen in Fig. 7) had the presence of a baseline characterised by a temporal drift and a DC-bias which is unrelated to the experiment being conducted. The baseline was corrected by fitting a 3rd-order polynomial using the procedure as described in [47].

Logical circuits were extracted from the recorded data through the threshold classification of measured pulses. A logical 1 was assigned to the respective pulse if it was outside the bipolar threshold band, and a logical 0 was assigned if it was inside. A total of 18 thresholds were considered with values ranging from 100 mV up to 1 V with steps of 50 mV.

**Fig. 8:**
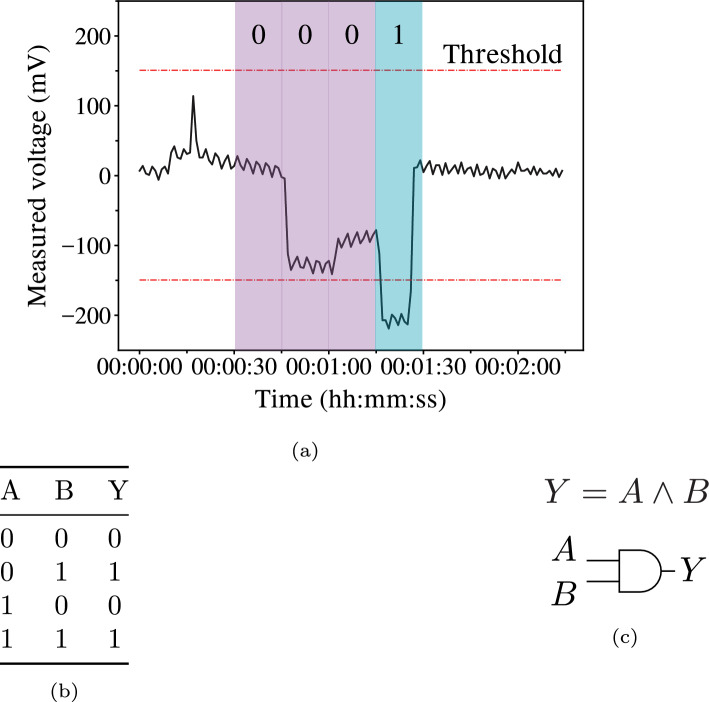
**a** Pulse classification for 2-bit strings, **b** Resulting truth table for classified pulses, and **c** extracted sum of products Boolean expression and realised logical circuit

The binary strings and respective classified pulses were stored in truth tables, and the minimal sum of products (SOP) Boolean expression was calculated for each truth table using the Quine–McCluskey algorithm [48, 49]. The logical circuit was then realised from the calculated SOPs. In Fig. 8, the workflow for the pulse classification and logical circuit extraction used in this work for a 2-bit string input is shown.

Table 1 shows the most frequently extracted sum of products (SOP) Boolean expression for 2-bit string inputs. The consistent appearance of the $A \vee B$ form, observed 151 times throughout the analysis, indicates a complex OR logic transduction within the colloid substrate when subjected to multidimensional input stimuli.

**Table 1:** Most frequent extracted sum of products (SOP) Boolean expression for 2-bit string inputs with thresholds varying from 50 mV to 1 V with 50 mV steps

SOP	Count
$A\vee B$	151
$A\wedge \lnot B$	79
$(A\wedge \lnot B) \vee (\lnot A\wedge B)$	52
$A\wedge B$	2

**Fig. 9:**
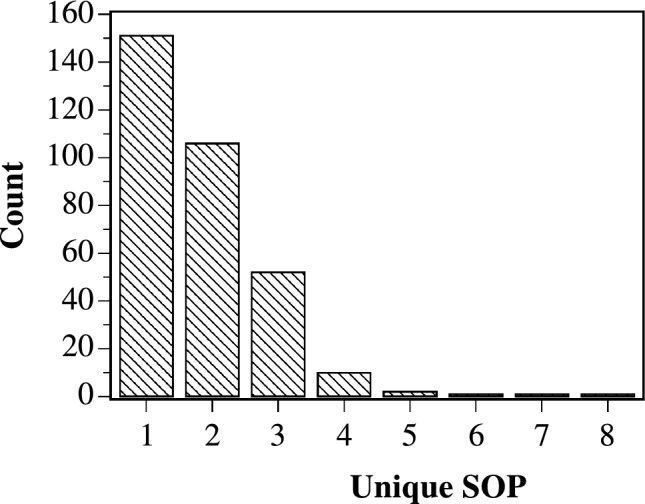
Distribution of different Sum-of-Products (SOP) Boolean logic operations exhibited by a proteinoid–ZnO-based system with 2-bit binary strings

A Proteinoid ZnO-based system’s distribution of various SOP Boolean logic operations is depicted in Fig. 9. The horizontal axis corresponds to the different SOP motifs found, and the vertical axis shows the frequency of each SOP logic operation over potential 2-bit input strings. The inclusive OR function ($A \vee B$) is the most common logic gate, occurring 151 times, suggesting that the system has an inherent preference for disjunctive logic.

**Fig. 10:**
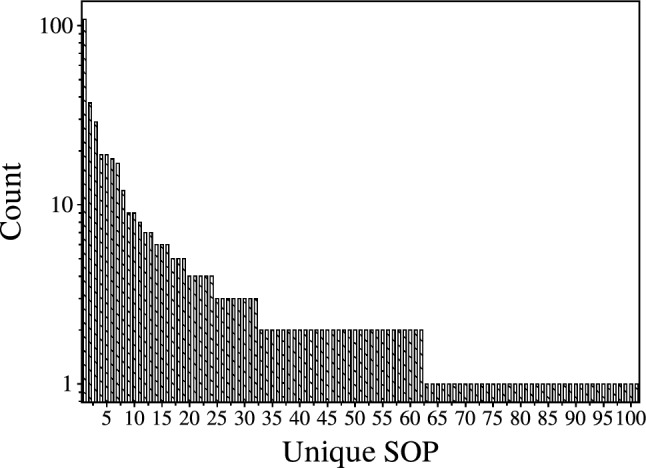
The occurrence frequency of the most common SOP Boolean logic operations performed by the ZnO–proteinoid system with 4-bit string inputs

Figure 10 visualises the frequency of the most common SOP Boolean logic operations executed by the ZnO–proteinoid system when presented with all possibilities of 4-bit string inputs. The inclusive OR function, which involves all variables, was the most prevalent, occurring 108 times. This reflects the significant presence of disjunctive logic within the system. Other operations and higher-order combinations were also frequently observed. These findings suggest a wide range of computational flexibilities within our system, capable of handling complex, multidimensional data.

**Table 2:** Extracted SOP Boolean expression for 4-bit string inputs with varying thresholds for the ZnO–proteinoids system

SOP	Count
$A \vee B \vee C \vee D$	108
$(A \wedge C \wedge \lnot B \wedge \lnot D) \vee (B \wedge D \wedge \lnot A \wedge \lnot C)$	37
$(A \wedge \lnot B \wedge \lnot D) \vee (B \wedge \lnot C) \vee (A \wedge C \wedge \lnot B) \vee (B \wedge D \wedge \lnot A) \vee (C \wedge \lnot B \wedge \lnot D)$	29
$(A \wedge C \wedge \lnot B \wedge \lnot D) \vee (B \wedge \lnot A \wedge \lnot C)$	19

Table 2 depicts extracted SOP Boolean expression for 4-bit string inputs with varying thresholds for the ZnO–proteinoids system. The most common SOP logic operations obtained from the ZnO–proteinoids system when given all conceivable 4-bit binary input combinations. The dominant emergent of OR function, 108 times, confirms the widespread presence of disjunctive logic. Moreover, Other operations and higher-order combinations were frequently observed, suggesting a sophisticated and varied logic transduction that encompasses multidimensional inputs. By describing the modular logic patterns that emerge from interactions in non-von Neumann substrates, we can derive fundamental principles for deliberately designing desired computations.

**Table 3:** Most frequent extracted sum of products Boolean expression for an 8-bit string input with varying thresholds for the ZnO–proteinoids system

SOP	Count
$(A \wedge \lnot E) \vee (B \wedge \lnot H) \vee (C \wedge \lnot G) \vee (D \wedge \lnot F) \vee (E \wedge \lnot D) \vee (F \wedge \lnot C) \vee (G \wedge \lnot B) \vee (H \wedge \lnot A)$	8
$(B \wedge \lnot F) \vee (C \wedge \lnot E) \vee (D \wedge \lnot C) \vee (E \wedge \lnot B) \vee (F \wedge \lnot A) \vee (G \wedge \lnot D) \vee (H \wedge \lnot D) \vee (A \wedge \lnot G \wedge \lnot H)$	4
$(A \wedge \lnot D) \vee (B \wedge \lnot G) \vee (C \wedge \lnot F) \vee (D \wedge \lnot E) \vee (E \wedge \lnot C) \vee (F \wedge \lnot B) \vee (G \wedge \lnot A) \vee (H \wedge \lnot A)$	3
$(A \wedge \lnot E) \vee (C \wedge \lnot G) \vee (D \wedge \lnot F) \vee (E \wedge \lnot D) \vee (F \wedge \lnot C) \vee (G \wedge \lnot B) \vee (H \wedge \lnot E) \vee (B \wedge \lnot A \wedge \lnot H)$	2

Table 3 presents the most frequent sum-of-product expressions identified through analysis of all 256 possible input combinations from the proteinoid–ZnO colloidal system, characterised by 8-bit string inputs. As we investigated higher dimensions, we discovered that the system’s fundamental logic favoured particular patterns.

**Fig. 11:**
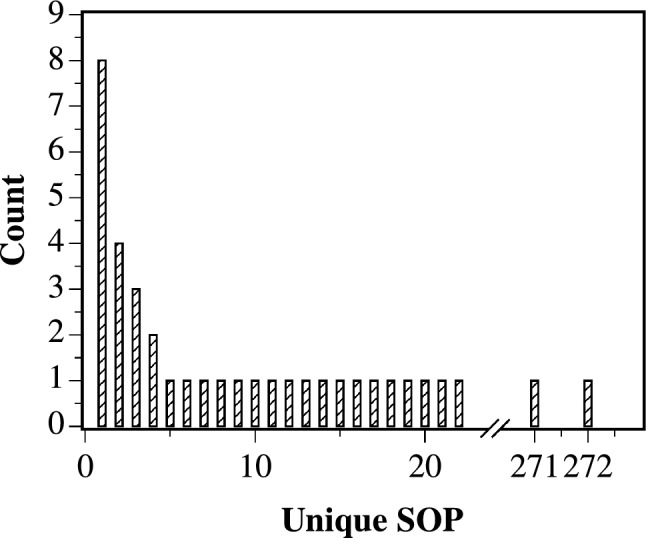
The most frequently encountered SOP Boolean logic expressions were determined through a comprehensive analysis of 8-bit binary string inputs within the ZnO–proteinoids system

The bar chart in Fig. 11 presents a comparative study of the most common SOP Boolean logic expressions found in the ZnO–proteinoids system after all 256 potential combinations of 8-bit binary string inputs were examined in detail. The *y*-axis displays the frequency of each expression, and the *x*-axis lists the expressions that occur most frequently. With eight occurrences, an equality function emerged as the dominating function, which is appropriate for dealing with higher-dimensional data. This plot highlights intrinsic processing capability by displaying a list of the system’s favourite logic operations that it naturally uses when dealing with a variety of biological and inorganic inputs.

### Fault tolerance

Neuromorphic computing is quickly gaining widespread acceptance, with Resistive Random Access Memory (RRAM) and RRAM-based computing systems (RCS) emerging as promising hardware implementations for neuromorphic computing. The immature fabrication process poses a challenge for RCS, making it prone to defects that can cause substantial accuracy reductions in neuromorphic computing applications [35]. The techniques suggested in the literature to address stuck-at-faults involve extra operations and circuit overhead, leading to increased power consumption and delays [50].

**Fig. 12:**
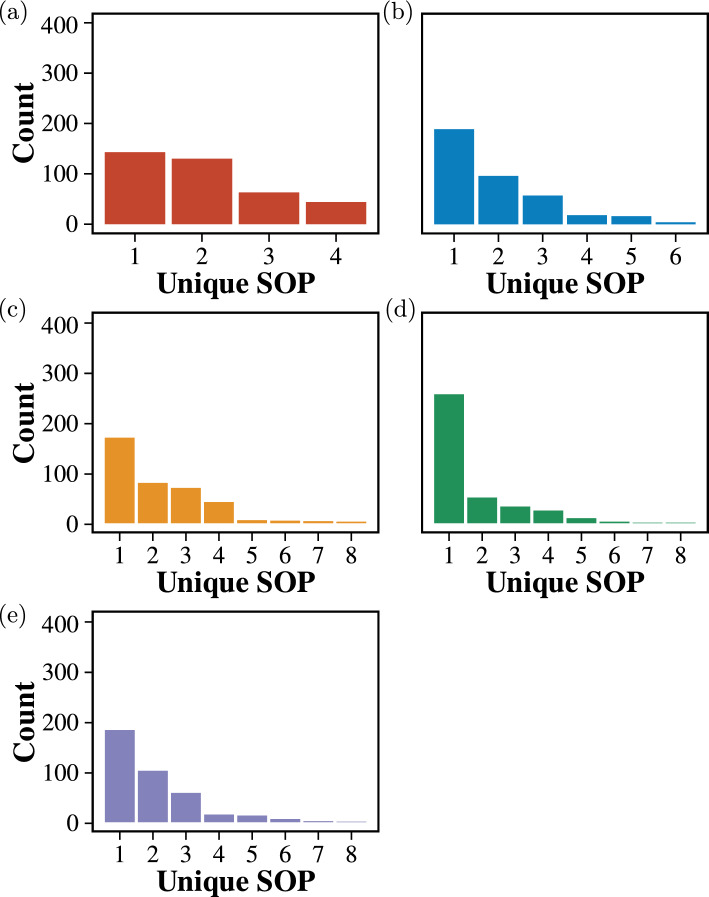
The most frequently encountered SOP Boolean logic expressions were determined through a comprehensive analysis of 2-bit binary string inputs within the ZnO–proteinoids system with **a** 3 ml, **b** 3.5 ml, **c** 4 ml, **d** 4.5 ml, and **e** 5 ml volume of the mixture

**Fig. 13:**
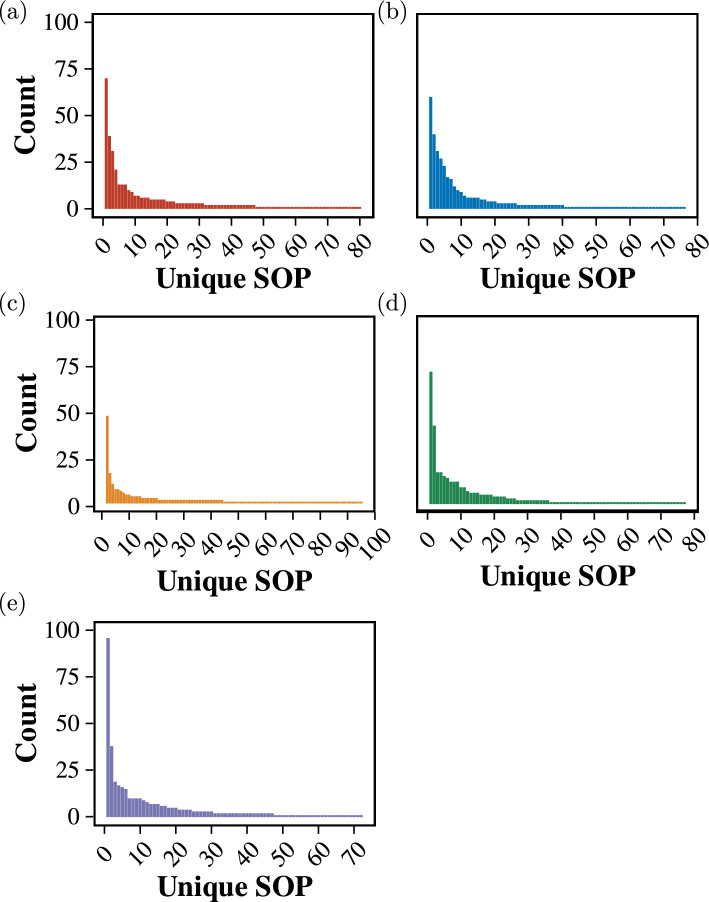
The most frequently encountered SOP Boolean logic expressions were determined through a comprehensive analysis of 4-bit binary string inputs within the ZnO–proteinoids system with **a** 3 ml, **b** 3.5 ml, **c** 4 ml, **d** 4.5 ml, and **e** 5 ml volume of the mixture

To find the fault tolerance ability of the Proteinoid and ZnO colloid mixture system, we repeated the previous experiments but changed the volume of the mixture for each trial. Figure 12 depicts the most frequently encountered SOP Boolean logic expressions that were determined from 2-bit binary string inputs within the ZnO–proteinoids system with different volumes of the mixture, and Fig. 13 illustrates the same results for 4-bit binary string inputs.

**Fig. 14:**
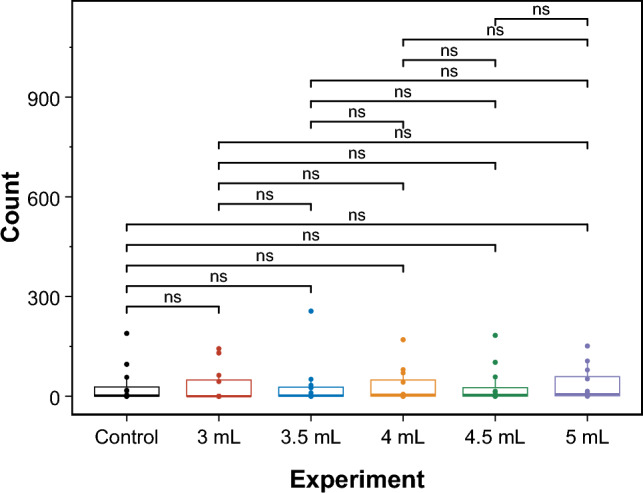
Box plot of pairwise comparisons for the extracted SOP for the ZnO–proteinoids system with 3 ml, 3.5 ml, 4 ml, 4.5 ml, and 5 ml and Control for 2-bit strings input. The symbol ns indicates the statistical significance of the test ($p\ge 0.05$)

**Fig. 15:**
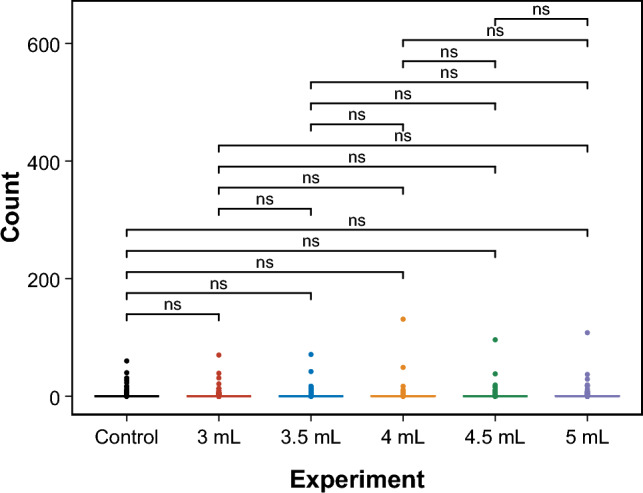
Box plot of pairwise comparisons for the extracted SOP for the ZnO–proteinoids system with 3 ml, 3.5 ml, 4 ml, 4.5 ml, and 5 ml and Control for 4-bit strings input. The symbol ns indicates the statistical significance of the test ($p\ge 0.05$)

To compare the distributions of unique SOP between the original measurements (referred to as “control”), we used the pairwise Wilcoxon signed-rank test [51]. The test for 2- and 4-bit experiments is illustrated by the box plots shown in Figs. 14 and 15, respectively. The figures also display the calculated *p* values ($\ge$ 0.05, denoted as ns for non-significant) for all performed tests. These *p* values indicate that the null hypothesis, which posits no significant difference between the paired observations in the population, cannot be rejected. Thus, it can be concluded that the observed logic functions do not exhibit any statistically significant changes even with changes in volume. This indicates that the system is highly robust, with a high tolerance for faults caused by such variations. These findings illustrate the system’s reliability under volumetric change, emphasising its promise for practical applications where fault tolerance is crucial.

Our laboratory investigations revealed that a wide range of many-input logical gates may be implemented inside a colloidal mixture of ZnO nanoparticles and proteinoids. We found that the two-input, four-, and eight-input functions exhibited significant nonlinearity, implying that the dynamical behaviour of colloid-based logic devices may be influenced by many attractors and branching points in addition to resistive switching.

While the precise mechanisms underlying the reported Boolean frequency profiles are ambiguous, we propose that specific physicochemical characteristics of the colloidal system may elucidate the frequency distribution in these logic gates. Particle interaction and aggregation, where stable aggregates under particular electrical conditions impact gate creation, may be the initial reason. Chemical functional groups on particle surfaces may facilitate particular gates, analogous to previously reported reactions between ethoxylated groups and oxygen vacancies in ZnO nanoparticles. Surface chemistry may potentially be involved. Through charge distribution and field strength, electric field effects may have an additional impact on gate creation by affecting particle mobility and interaction [44].

The fault tolerance of colloid-based computing arises from the amorphous characteristics of the colloidal mixture, which demonstrated exceptional adaptability and reconfigurability when subjected to electrical stimulation, generating spikes of electrical potential. These findings highlight the viability of colloid-based computing for future unconventional computing technologies.

Future studies will focus on reprogramming colloid computers via modifications to particle configurations, such as varying surface chemistry, modifying electric or magnetic fields, or modifying particle size. Scaling up colloid computers through parallel processing, fine-tuning manipulation accuracy, and particle count increases are other significant fields. Further improvements in computing power could be made by modifying input signals to accomplish in-line execution or cascading colloid droplets to create multi-level logical circuits. Introducing innovative computer possibilities may also require the introduction of new physical concepts, such as quantum interactions [45].

Our current discoveries establish the framework for these objectives, as colloids show promise for in-memory computing, with many Boolean functions indicating a pre-built programme within the system. Subsequent research endeavours will enhance the modulation of input signals and investigate additional applications in neuromorphic computing, where low-duty cycles could yield spike-based inputs. These developments are expected to improve colloid-based computer systems’ capabilities and applicability in developing technologies.

## Conclusion

This research presents a novel approach that combines ZnO colloids and proteinoids in a dynamic colloidal mixture to implement Boolean logic functions. The synergy between ZnO nanoparticles and the proteinoid matrix enables the system to act as a fault-tolerant, reconfigurable logic gate, responding to electrical impulses with consistent and reproducible logic functions. The computational abilities observed in this system are underpinned by the formation of electrostatic dipoles within the proteinoid matrix and their interactions with ZnO nanoparticles, which introduce anisotropy and stabilize the system’s response to external signals.

Through a series of experiments, we demonstrate that this system can act as a fault-tolerant, reconfigurable logic gate, responding to electrical impulses with consistent logic functions. Moreover, the orientation effects of ZnO nanoparticles under applied electric fields and their role in governing the structural and electrical properties of the colloidal mixture offer new insights into controlling and enhancing the performance of hybrid computational systems.

The results demonstrate the feasibility of using nano-bio hybrid systems for unconventional computing applications, particularly in scenarios requiring adaptability, fault tolerance, and low-energy operation. The ability of the ZnO–proteinoid system to exhibit robust computational functionality under varied experimental conditions highlights its potential in emerging fields such as neuromorphic computing, bio-inspired systems, and in-memory computing. The tunability of this system through the manipulation of its physical and chemical properties opens avenues for customizing its behaviour for specific computational tasks.

The findings of this research represent a significant step towards the development of unconventional computing systems that leverage the unique capabilities of nano-bio materials. The potential applications range from biosensors and low-power adaptive devices to scalable computational architectures, offering exciting prospects for the future of computing technologies.

## Data Availability

The datasets made and analysed during the current study are available from the corresponding author and also are uploaded to the Zenodo open repository (10.5281/zenodo.11080506).
